# Assessment of costs associated with adverse events in patients with cancer

**DOI:** 10.1371/journal.pone.0196007

**Published:** 2018-04-13

**Authors:** William Wong, Yeun Mi Yim, Ashley Kim, Martin Cloutier, Marjolaine Gauthier-Loiselle, Patrick Gagnon-Sanschagrin, Annie Guerin

**Affiliations:** 1 Health Economics and Outcomes Research, US Medical Affairs, Genentech, Inc., South San Francisco, California, United States of America; 2 Analysis Group, Inc, Montréal, Québec, Canada; Universidade do Algarve Departamento de Ciencias Biomedicas e Medicina, PORTUGAL

## Abstract

Adverse event (AE)-related costs represent an important component of economic models for cancer care. However, since previous studies mostly focused on specific AEs, treatments, or cancer types, limited information is currently available. Therefore, this study assessed the incremental healthcare costs associated with a large number of AEs among patients diagnosed with some of the most prevalent types of cancer. Data were obtained from a large US claims database. Adult patients were included if diagnosed with and treated for one of the following cancer types: breast, digestive organs and peritoneum, genitourinary organs (including bladder and ovary and other uterine adnexa), lung, lymphatic and hematopoietic tissue, and skin. Treatment episodes were defined as the period from initiation of the first antineoplastic pharmacologic therapy to discontinuation (i.e., gap of ≥ 45 days), or change in treatment regimen, or end of data availability. A total of 36 AEs were selected from the product inserts of 104 treatments recommended by practice guidelines. A retrospective matched cohort design was used, matching a treatment episode with a certain AE with a treatment episode without that AE. A total of 412,005 patients were selected, for a total of 794,243 treatment episodes, resulting in 1,617,368 matched treatment episodes across all 36 AEs. Incremental healthcare costs associated with AEs of any severity ranged from $546 for cough/upper respiratory infections to $24,633 for gastrointestinal perforation. The three most costly AEs when considering any severity were gastrointestinal perforation ($24,633), central nervous system hemorrhage ($24,322), and sepsis/septicemia ($23,510). Incremental healthcare costs associated with severe AEs ranged from $15,709 for dermatitis and rash to $48,538 for gastrointestinal fistula. The three most costly severe AEs were gastrointestinal fistula ($48,538), gastrointestinal perforation ($41,281), and central nervous system hemorrhage ($38,428). In conclusion, AEs during treatment episodes for cancer were frequent and associated with a substantial economic burden.

## Introduction

Given the large, and increasing, number of cancer treatments currently available, there exists a growing demand for evidence-based studies and economic models to more effectively inform healthcare and policy decisions, particularly amidst healthcare budget restrictions and rising cancer care costs [1, 2]. Such models typically evaluate and compare the costs, effectiveness, and safety profiles of existing therapeutic options. In several types of cancer, the cost of care has been found to increase substantially when patients experience adverse events (AEs) [3–7]. This is not surprising given that AEs can negatively impact both clinical outcomes and quality of life and disrupt treatment plans, often leading to therapy changes (such as dose delays or reductions), lower adherence, and even discontinuation [8–11]. Because of the potentially substantial contribution of AEs to the economic burden of managing cancer patients [6, 12, 13], a comprehensive assessment of the costs associated with cancer care should include not only the costs of cancer care but also the costs of AE management [8].

Whilst AEs clearly represent an important component of any economic model aimed at assessing the overall economic burden of cancer care [2], the lack of information on AE-related costs makes economic modeling challenging. Indeed, although clinical trials routinely assess the incidence and severity of treatment-related AEs, the costs associated with the management of AEs are rarely evaluated. As such, the AE-related costs needed to build economic models are typically obtained from real-world studies [5, 6]. However, published data on the real-world costs of AEs among cancer patients are limited. In particular, most previous studies investigating the costs of AEs among cancer patients are generally restricted to specific AEs, treatments, drug classes, or cancer types [3–7, 9, 10, 14–16]. Moreover, findings from these studies may be difficult to combine into a single economic model due to variations in the methodology used for different AEs and cancer types. Furthermore, estimates of AE-related costs among cancer patients are often limited to the costs directly associated with the clinical management of AEs, failing to represent the actual economic burden that may result from experiencing a certain AE during cancer treatment [3].

Therefore, the objective of this study was to estimate the incremental healthcare costs associated with a large number of AEs among patients diagnosed with some of the most prevalent types of cancer, including cancer of the bladder, breast, digestive organs and peritoneum, genitourinary organs (excluding bladder, ovary and other uterine adnexa), lung, lymphatic and hematopoietic tissue, ovary and other uterine adnexa, and skin.

## Materials and methods

### Data source

Data were obtained from the Truven Health Analytics MarketScan® database (01/01/2006–09/30/2015), which includes enrollment history and claims for medical (provider and institutional) and pharmacy services for employees, their dependents, and Medicare-eligible retirees with employer-provided Medicare supplemental plans covered by the health benefit programs of large US employers. The MarketScan database is compliant with the Health Insurance Portability and Accountability Act and contains no identifiable patient information; thus, no institutional review board approval was necessary for this study.

### Patient selection

**Fig 1:**
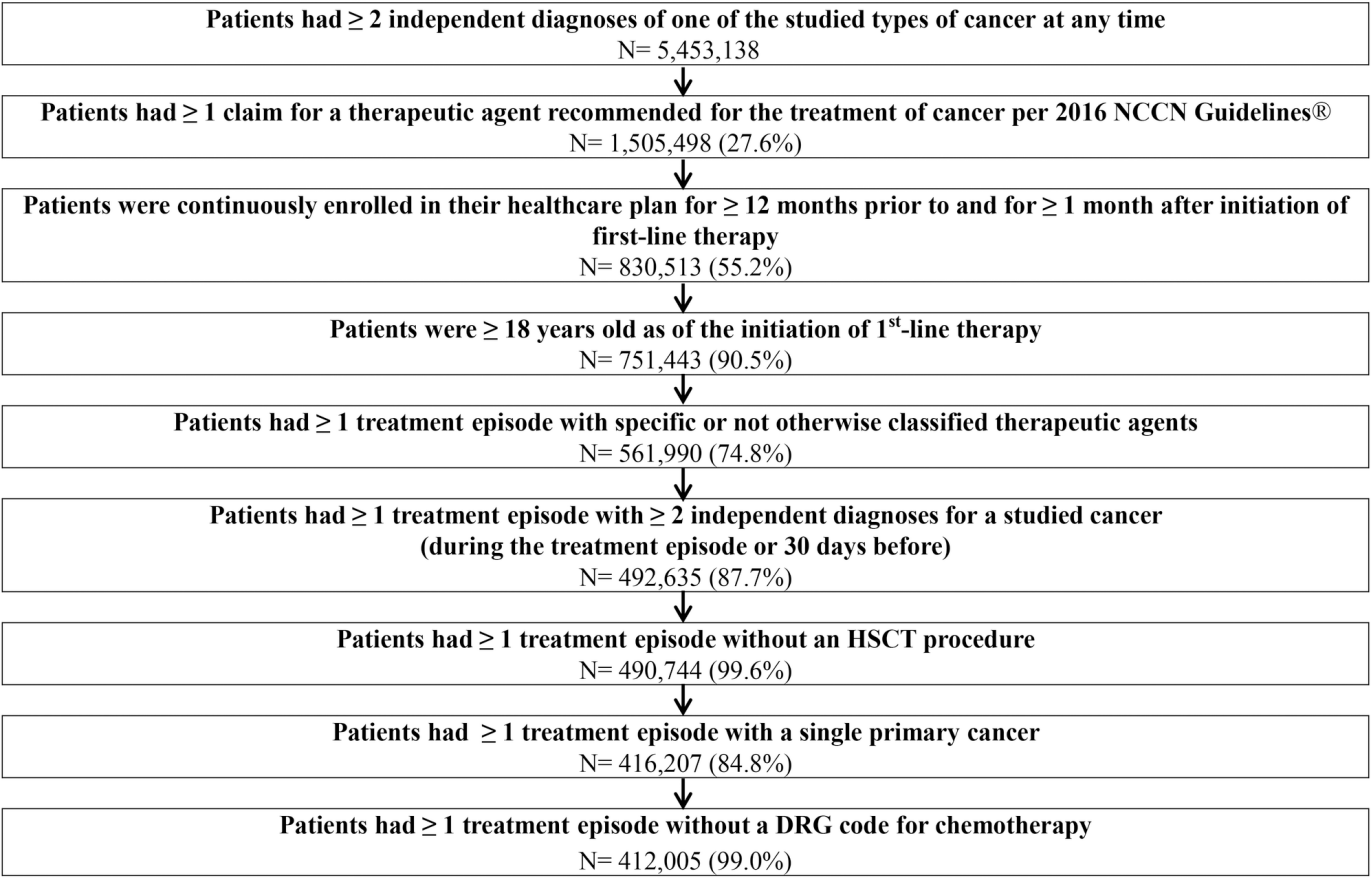
Patient selection. NCCN: National Comprehensive Cancer Network, HSCT: Hematopoietic Stem Cell Transplantation, DRG: Diagnosis-Related Group.

Patient selection is described in Fig 1. Adult patients were required to have been diagnosed and treated for one of the following types of cancer: bladder (International Classification of Diseases, Ninth Revision, Clinical Modification [ICD-9-CM] 188.xx); breast (ICD-9-CM 174.xx or 175.xx); digestive organs and peritoneum (ICD-9-CM 150.xx-159.xx); ovary and other uterine adnexa (ICD-9-CM 183.xx); genitourinary organs, excluding bladder, ovary and other uterine adnexa (ICD-9-CM 179.xx - 182.xx, 184.xx-187.xx, 189.xx); lung (ICD-9-CM 162.xx); lymphatic and hematopoietic tissue (ICD-9-CM 200.xx-208.xx); skin (ICD-9-CM 172.xx-173.xx, 232.xx).

### Study design

**Fig 2:**
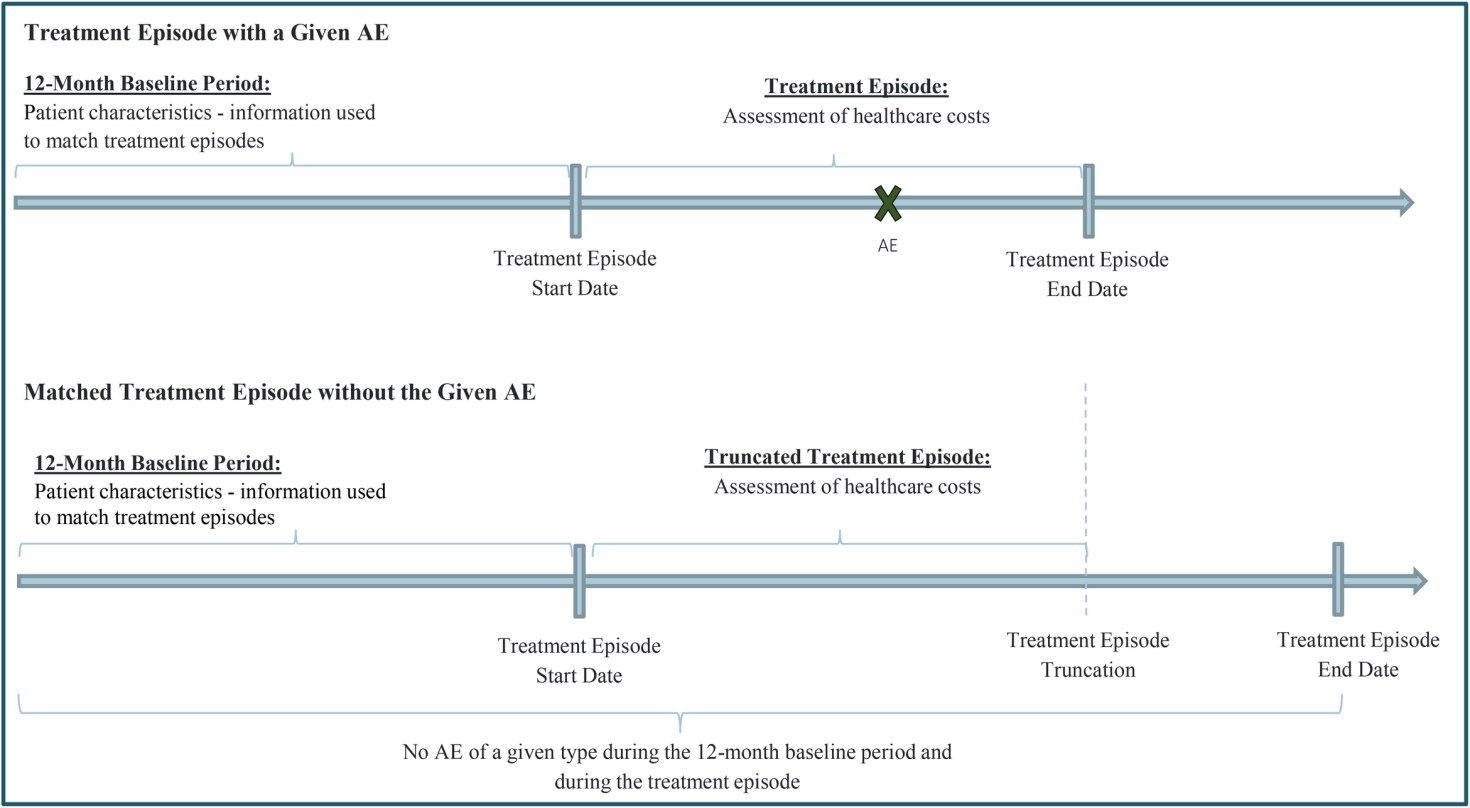
Study design. AE: adverse events.

A retrospective matched cohort design was used to assess the incremental costs associated with AEs during cancer treatment episodes, where treatment episodes with a certain AE were matched with treatment episodes without that AE, as detailed below. The *study index date* was defined as the initiation date of the first-line therapy for one of the types of cancer listed above. The *study baseline period* was defined as the 12-month period prior to the study index date, and served as a washout period to identify a patient’s first-line cancer therapy (Fig 2). On the other hand, the 12-month period prior to the start of each treatment episode was defined as *episode baseline period* and served to determine the patient characteristics to be used for the matching of treatment episodes and for multivariate adjustments (Fig 2). Lastly, the *study period* was defined as the period spanning from the study index date to the end of data availability or continuous health plan enrollment, whichever occurred first.

### Treatment episodes

A treatment episode was defined as the period spanning from the initiation of the first antineoplastic pharmacologic agent, as part of a treatment regimen, to the first of the following events: (1) discontinuation of the treatment regimen (i.e., a gap of ≥ 45 days in the use of all the agents that were part of the treatment regimen); (2) a change in treatment regimen (i.e., substitution or addition of an antineoplastic pharmacologic agent to the treatment regimen; the addition of radiotherapy and the discontinuation of a single agent from a combination therapy were not considered as constituting a change in treatment regimen); (3) the end of continuous health plan enrollment; or (4) the end of data availability. Treatment regimens were identified based on all the antineoplastic pharmacologic agents used during the first 28 days after initiating a new antineoplastic pharmacologic agent.

For eligible patients, all treatment episodes meeting the inclusion and exclusion criteria (Fig 1) were selected for the analyses. Thus, a patient could be included in the analysis more than once at different times (e.g., once during first-line therapy and once during second-line therapy).

### Classification and matching of treatment episodes

Eligible treatment episodes were classified into two cohorts: *treatment episode with AE* (i.e. a diagnosis of a certain AE was recorded during the treatment episode), and *treatment episode without AE* (i.e., a diagnosis for that same AE was not recorded during the entire duration of the treatment episode *and* during the 12-month period preceding the start date of the treatment episode).

In order to identify AEs to be considered in this study, tumor-specific National Comprehensive Cancer Network Clinical Practice Guidelines were first reviewed and 104 recommended treatments were identified for the types of cancer included in the study. A list of 361 AEs was identified from the product inserts of these treatments. AEs were further combined and/or selected to be included in the study based on the frequency of their mention in the product inserts, their relevance (e.g., maximum grade severity 3 or 4), and availability of their respective ICD-9-CM diagnosis codes, such that a total of 36 AEs associated with these treatments were studied.

AEs of any severity were defined based on the presence of a diagnosis (ICD-9-CM codes) recorded on a health insurance claim in any medical setting (e.g., inpatient, emergency room, outpatient). Severe AEs were defined as AEs recorded on a health insurance claim for an inpatient stay. Treatment episodes with a certain AE were matched to similar treatment episodes without that same AE on a 1:1 ratio based on the patient’s age, gender, region of residence, type of health plan, type of primary cancer, metastatic status (based on secondary malignant diagnoses ICD-9-CM: 196.x, 197.x, 198.x, 199.0, 199.1), cancer-related surgery in the 3-month period prior to or during the treatment episode, type of treatment regimen, and line of therapy.

To compare treatment episodes with and without AE over the same duration, treatment episodes with AE were first matched to treatment episodes without AE that had the same or longer duration (treatment episodes without AE are expected to last longer than similar treatment episodes with AE because patients are less likely to change or discontinue treatment); subsequently, the duration of the treatment episode without AE was truncated to be equal to the duration of the matched treatment episode with AE (Fig 2).

The matching of treatment episodes was performed separately for each AE. As such, while treatment episodes could be matched only once for a given AE, they could be matched multiple times across AEs. (e.g., if a patient experienced more than one AE during a certain treatment episode).

### Outcomes and statistical analyses

Healthcare costs were measured from a US payers' perspective (amount reimbursed by the commercial plan and coordination of benefits) and were defined as the sum of pharmacy and medical service costs (including inpatient, durable medical equipment, emergency room, and outpatient costs). Costs related to cancer therapies (e.g., chemotherapy administration costs, radiotherapy costs) were excluded from the analyses to avoid capturing differences in costs between cohorts that would be driven by differences in cancer therapy costs (e.g., adherence and dosing) and not associated with the studied AE.

Healthcare costs were assessed during the treatment episode and were reported in 2015 US dollars. Thus, healthcare costs included the costs of the initial management of a certain AE as well as all the subsequent costs resulting from that AE (e.g., follow-up visits or management of worsening symptoms due to treatment modifications).

Healthcare costs were compared between matched treatment episodes with and without a given AE using multivariate generalized linear models (GLM) with a log link and a gamma distribution. A separate regression was estimated for each AE. Healthcare costs were also stratified for each type of cancer. Analyses were adjusted for Charlson comorbidity index, year of treatment episode start, the presence of AEs other than the AE of interest during the studied treatment episode, and other potential confounders associated with important resource utilization (diabetes mellitus, fluid and electrolyte disorders, dehydration/diaphoresis, hyperlipidemia, renal failure, respiratory failure, and pulmonary circulation disorders).

Confidence intervals were calculated using non-parametric bootstrap estimation with 499 iterations.

## Results

### Characteristics across matched treatment episodes

The selected 412,005 patients (Fig 1) had a total of 794,243 treatment episodes, resulting in 1,617,368 matched treatment episodes across all 36 AEs (Table 1). Across matched treatment episodes, patients had a mean age of 61.8 years and 72.6% were females. The mean Charlson comorbidity index score was 4.1 and 57.7% of patients were receiving first-line therapy. The most frequent primary cancer was breast cancer (58.7%), followed by cancer of genitourinary organs, excluding bladder and ovary and other uterine adnexa, (10.7%), digestive organs and peritoneum (8.6%), lymphatic and hematopoietic tissue (8.6%), lung (7.8%), bladder (3.2%), ovary and other uterine adnexa (1.3%), and skin (1.1%).

**Table 1:** Patient characteristics across treatment episodes.

Patient Characteristics	All Treatment Episodes	Matched Treatment Episodes^a^
	N = 794,243	N = 1,617,368
**Age, Years; Mean ± SD [Median]**	**62.8 ± 13.2 [62]**	**61.8 ± 12.4 [61]**
**Female, N (%)**	**461,818 (58.1%)**	**1,174,222 (72.6%)**
**Charlson Comorbidity Index, Mean ± SD [Median]**	**4.4 ± 2.4 [4]**	**4.1 ± 2.3 [3]**
**Line of Therapy, N (%)**		
1^st^ Line	360,016 (45.3%)	933,732 (57.7%)
2^nd^ Line	192,779 (24.3%)	392,462 (24.3%)
3^rd^ Line +	241,448 (30.3%)	291,174 (18.0%)
**Type of Studied Cancer**		
Breast	315,547 (39.7%)	948,802 (58.7%)
Digestive Organs and Peritoneum	93,865 (11.8%)	138,946 (8.6%)
Lung	66,984 (8.4%)	126,724 (7.8%)
Lymphatic and Hematopoietic Tissue	72,210 (9.1%)	138,854 (8.6%)
Skin	16,389 (2.1%)	18,060 (1.1%)
Bladder	40,484 (5.1%)	51,646 (3.2%)
Ovary and Other Uterine Adnexa	15,378 (1.9%)	21,250 (1.3%)
Other Genitourinary Organs	173,386 (21.8%)	173,086 (10.7%)
**Metastatic Cancer, N (%)**	**206,460 (26.0%)**	**259,578 (16.0%)**
Visceral Metastases	98,066 (12.3%)	80,738 (5.0%)
Bone and Bone Marrow Metastases	73,707 (9.3%)	64,296 (4.0%)
Central Nervous System Metastases	22,532 (2.8%)	10,396 (0.6%)
Lymph Nodes Skin and Other Metastases	88,552 (11.1%)	136,638 (8.4%)
Disseminated Cancer	954 (0.1%)	80 (0.0%)
**Surgery within Prior 3 Months, N (%)**	**171,806 (21.6%)**	**444,852 (27.5%)**
**Surgery During Treatment Episode, N (%)**	**63,233 (8.0%)**	**157,518 (9.7%)**

[a] A treatment episode could be matched only once for a given AE, but could be matched multiple times across all AEs.

When considering treatment episodes by AE, the number of treatment episodes matched across AEs ranged from 878 to 115,754 (**Table 2**). Among matched treatment episodes with AE of any severity, the most prevalent AEs were pain (28.2%), hypertension (27.5%), anemia/pallor (17.8%), psychiatric disorders (13.9%), and cough/upper respiratory infections (13.6%) (Table 2). Among all AEs, sepsis/septicemia was the AE with the largest proportion of events considered severe (i.e., recorded during an inpatient stay) (79.6%; Table 2). Lastly, the mean duration of matched treatment episodes ranged from 4.7 to 16.4 months (Table 2).

**Table 2:** Prevalence and characteristics of matched treatment episodes by AE.

Adverse Event	Proportion of Episodes with AE of any severity^a^ (among all episodes)	Number of matched episodes	Proportion of matched episodes with severe AE^b^	Mean duration of matched episodes (months)
Pain	28.2%	115,754	10.9%	7.0
Hypertension	27.5%	118,890	10.1%	8.2
Anemia / Pallor	17.8%	112,744	13.6%	7.9
Psychiatric Disorders	13.9%	122,390	10.2%	10.0
Cough / Upper Respiratory Infections	13.6%	103,526	6.1%	11.1
Dyspnea	12.0%	79,724	26.5%	9.6
Neutropenia / Leukopenia	11.6%	84,300	12.3%	4.7
Vomiting	10.5%	76,952	11.3%	5.5
Chest Pain / Angina	10.3%	78,464	23.1%	11.0
Thromboembolic Events	8.6%	62,326	19.9%	8.9
Arrhythmia	8.5%	64,100	25.5%	8.4
Dysphagia / Esophagitis / Dyspepsia	7.6%	66,616	11.0%	12.4
Cystitis / Urinary Tract Infections	7.4%	65,964	11.8%	12.2
Hypothyroidism / Hyperthyroidism	7.1%	70,132	10.5%	12.7
Retinal / Corneal / Sclera Problems	4.9%	38,206	0.6%	13.6
Diarrhea	4.7%	36,682	20.2%	11.0
Generalized Edema	4.6%	36,578	9.7%	11.7
Pneumonitis / Pneumonia	4.4%	29,768	52.6%	8.3
Heart Failure	4.0%	31,556	37.4%	8.0
Constipation	3.3%	25,798	10.9%	11.1
Haematuria	3.1%	26,618	4.9%	11.1
Nausea Alone	3.1%	26,298	6.0%	7.1
Neuropathy / Peripheral Neuropathy	3.1%	25,646	5.2%	10.7
Thrombocytopenia	3.0%	18,002	27.3%	6.5
Sepsis / Septicemia	2.5%	16,014	79.6%	5.7
Dermatitis and Rash	2.4%	20,668	4.4%	12.9
Hypotension	2.1%	15,134	42.8%	7.6
Gastrointestinal Bleeding	1.8%	14,478	32.6%	12.1
Allergic / Administration Site Reaction	1.5%	12,836	14.5%	10.9
Pruritus / Erythema	0.8%	8,000	1.8%	16.4
Stomatitis and Mucositis	0.8%	6,538	14.0%	8.4
Colitis	0.3%	2,936	20.1%	12.5
Central Nervous System Hemorrhage	0.2%	916	61.8%	9.2
Gastrointestinal Perforation	0.2%	998	59.7%	8.0
Gastrointestinal Fistula	0.1%	938	30.9%	7.3
Pancreatitis	0.1%	878	46.2%	8.8

AE: adverse events

[a] AEs of any severity were defined as AEs recorded on a health insurance claim in any medical setting (e.g., inpatient stay, emergency room visit, or outpatient visit)

[b] Severe AEs were defined as AEs recorded on a health insurance claim for an inpatient stay.

### Healthcare costs

**Fig 3:**
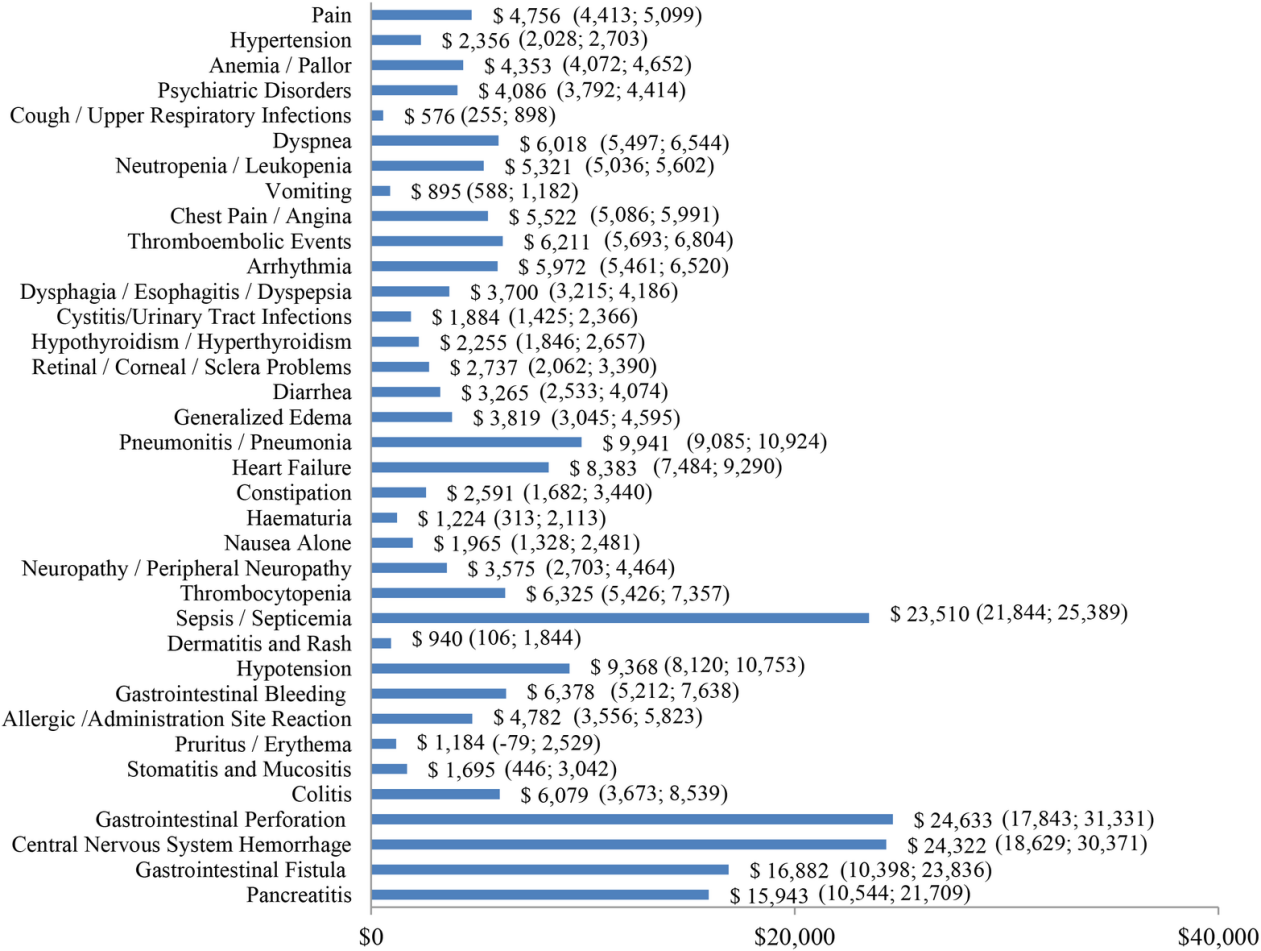
Incremental costs per treatment episode—AEs of any severity. AE: adverse events; 95% confidence intervals are presented in parentheses.

Incremental healthcare costs associated with AEs of any severity ranged from $576 for cough/upper respiratory infections to $24,633 for gastrointestinal perforation (Fig 3). The five most costly AEs of any severity were gastrointestinal perforation ($24,633), central nervous system hemorrhage ($24,322), sepsis/septicemia ($23,510), gastrointestinal fistula ($16,882), and pancreatitis ($15,943) (Fig 3).

**Fig 4:**
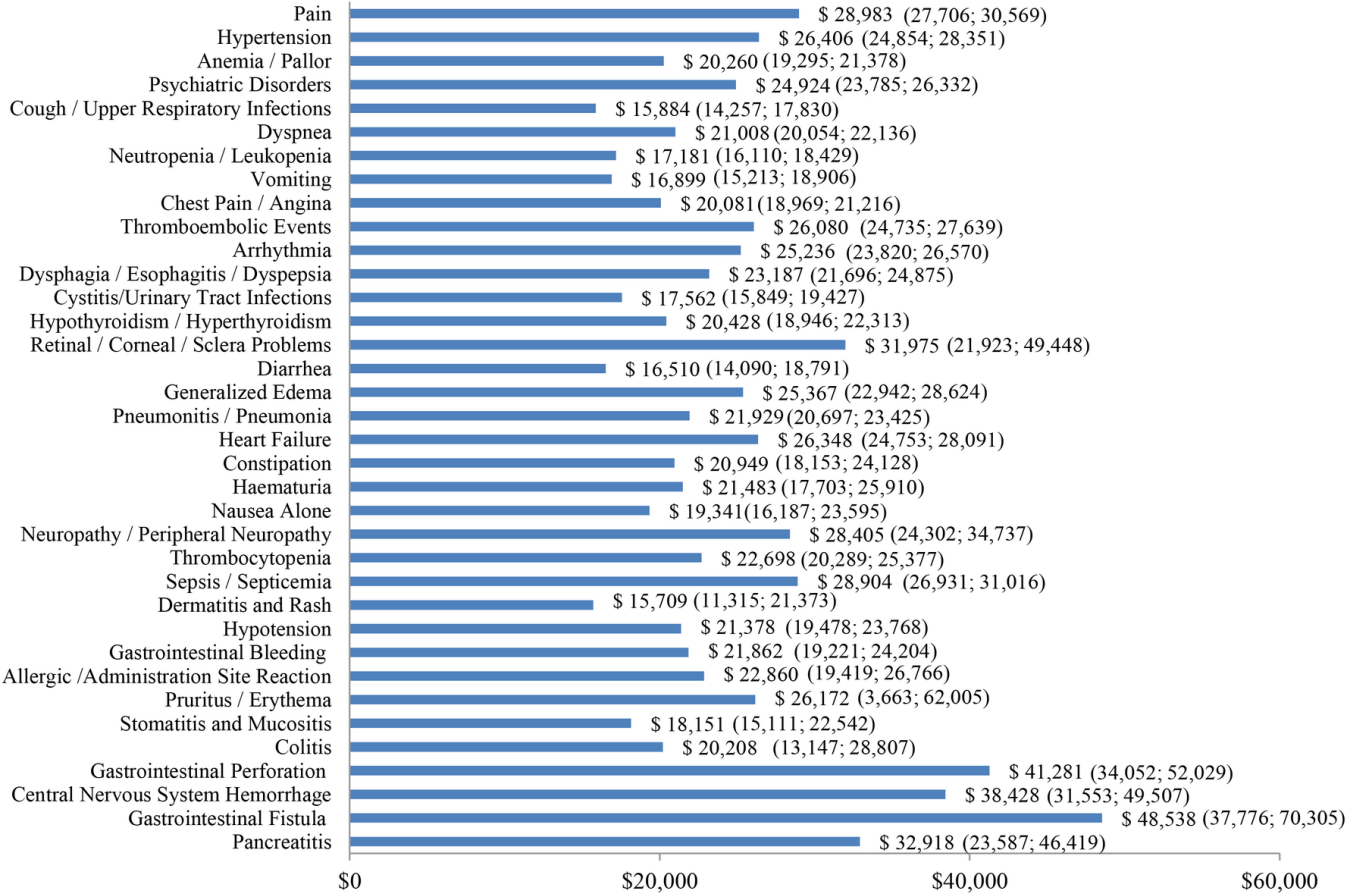
Incremental costs per treatment episode—severe AEs. AE: adverse events; 95% confidence intervals are presented in parentheses.

Incremental healthcare costs associated with severe AEs ranged from $15,709 for dermatitis and rash to $48,538 for gastrointestinal fistula (Fig 4). The five most costly severe AEs were gastrointestinal fistula ($48,538), gastrointestinal perforation ($41,281), central nervous system hemorrhage ($38,428), pancreatitis ($32,918), and retinal/corneal/sclera problems ($31,975) (Fig 4).

**Fig 5:**
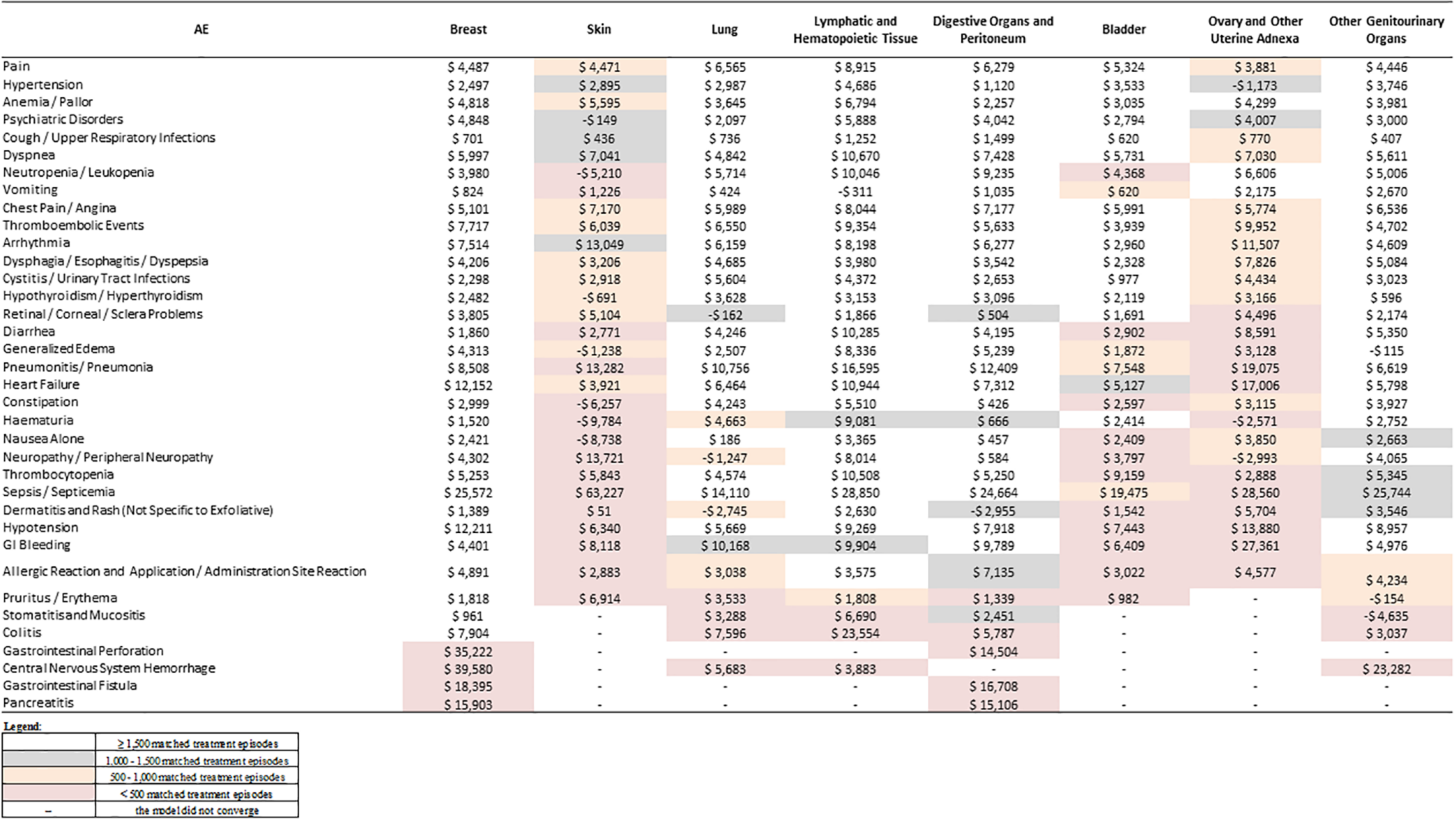
Increment costs of AEs, by cancer type. AE: adverse events.

For cancer types and AEs with sufficient sample size (N ≥ 1,500), the incremental healthcare costs associated with the most prevalent AEs were generally similar across cancer types, although some differences were observed (Fig 5). The costs associated with pain ranged from $4,446 to $ 6,565 across types of cancer except for cancer of lymphatic and hematopoietic tissue, for which the pain-related cost was $8,915. The costs associated with hypertension ranged from $ 2,497 to $ 3,746 across types of cancer except for cancer of digestive organs and peritoneum ($1,120) and lymphatic and hematopoietic tissue ($4,686). The costs associated with anemia/pallor ranged from $ 3,035 to $ 4,818 across types of cancer except for cancer of digestive organs and peritoneum ($2,257) and lymphatic and hematopoietic tissue ($6,794). The costs associated with psychiatric disorders ranged from $ 2,794 to $ 4,848 across types of cancer except for lung cancer ($2,097) and cancer of lymphatic and hematopoietic tissue ($5,888). Lastly, the costs associated with cough/upper respiratory infections ranged from $407 to $1,499 across all types of cancer.

## Discussion

Using a retrospective matched cohort design, this real-word study assessed the incremental healthcare costs associated with a large number of AEs among patients diagnosed with some of the most prevalent cancer types in the US.

The results of this study showed that AE-related healthcare costs were substantial. The five most costly AEs were gastrointestinal perforation ($24,633), central nervous system hemorrhage ($24,322), sepsis/septicemia ($23,510), gastrointestinal fistula ($16,882), and pancreatitis ($15,943). When stratified by type of cancer, the incremental healthcare costs associated with the most prevalent AEs were generally comparable, with some of the observed variations likely arising from differences in patient characteristics and the varying degree of AE severity across cancer types.

The methodology used here was based on macro-costing, which reports average costs for overall medical services [17]. Such approach was selected because it allows for an estimate of AE-related costs that goes beyond the AE management [18]. Another approach often employed to estimate real-world costs is micro-costing, in which cost estimates are derived from individual components of medical services [19]. Because of its dependence on individual rather than average data, micro-costing is useful to assess the costs of a new intervention or within-procedure variations, but typically provides only the costs incurred by a certain patient population at one or a small number of facilities, thus resulting in limited generalizability [17, 19]. Previous studies have found large differences (9% to 66%) in the estimated costs of care based on micro-costing and macro-costing approaches [20, 21]. In the case of AEs, given that micro-costing does not capture costs that are indirectly associated with AEs (e.g., costs resulting from potential treatment delay/disruption), macro-costing is often preferred when assessing AE-related costs.

Indeed, several studies have used a similar approach (the macro-costing approach) to estimate the economic burden of AEs among cancer patients for a variety of AEs. However, contrary to the current study, which included multiple cancer types, these prior studies mostly focused on one type of cancer at a time. For instance, Hurvitz et al. [6] estimated the healthcare costs of common AEs among patients with metastatic breast cancer, Latremouille-Viau et al. [5] among patients with metastatic colorectal cancer, and Arondekar et al. [16] and Bilir et al. [7] among patients with metastatic melanoma. On the other hand, a few other studies looked at healthcare costs across multiple cancer types, but were restricted, unlike this study, to specific AEs, often considering one AE at a time. For example, Weycker et al. [3, 22] estimated the healthcare costs of neutropenic complications of chemotherapy, while Pike et al. those of chemotherapy-induced peripheral neuropathy in patients with breast, ovarian, head and neck, and non-small cell lung cancer [4]. Some of these prior studies and others have also limited their estimates to the costs incurred during the initial management of AEs [7, 9]; this most likely resulted in underestimating the overall economic burden of AE given that other post-initial-management costs—such as those deriving from follow-up care, AE recurrence, and modification of the initial cancer treatment regimen—may also substantially contribute to increasing AE-related healthcare costs. As a case in point, Weyker et al. [3, 22] found that only 60% to 70% of the total neutropenia-related healthcare costs were attributable to the initial management of neutropenia, while 30% to 40% were attributable to follow-up care and subsequent neutropenia episodes.

Altogether, these results point to the need for a more comprehensive estimate of the healthcare costs related to the wide range of AEs that patients experience during antineoplastic treatment—one that includes the costs incurred both during and after the initial management of AEs. Importantly, whilst treatment effectiveness should always play a central role in guiding therapeutic decisions, the results of this study highlight the importance of also considering potential AEs when evaluating treatment options given the positive impact that fewer and less severe AEs may have on clinical outcomes and treatment adherence and persistence as well as healthcare costs. This is particularly important in light of the currently high costs of cancer care in the US. Indeed, in 2010, the costs of cancer diagnosis and management were estimated at 125 billion USD, representing approximately 5% of the annual US healthcare expenditures [23]. By 2020, this figure is projected to rise to 158–173 billion USD (in 2010 USD) [23]. Amidst these growing costs, economic models play an increasingly central role in informing healthcare and policy decisions [24]. For example, the Center for Medicare & Medicaid Services has developed the Oncology Care Model (OCM), a value-based reimbursement model aimed at incentivizing high-quality coordinated care (potentially including a reduction in AE-related hospitalizations and emergency department visits). Such models rely on estimates of real-world costs associated with cancer care. In addition, based on the International Society for Pharmacoeconomics and Outcomes Research (ISPOR) budget impact analysis good practices [25], costs associated with the management of AEs or complications should be considered in the costs of an intervention, and real-world studies provide a valuable source of information to be used in models when available. In line with the increasing need for cancer care cost estimates, this study provides much-needed information on the economic burden of a wide range of AEs across some of the most prevalent types of cancer in the US.

This study is subject to common limitations of retrospective observational studies based on healthcare claims data. First, since claims data contain limited information on the underlying cause of a given diagnosis, it was not possible to determine whether AEs were directly related to a treatment or underlying disease. Second, since AEs were identified based on recorded diagnoses and no information on grade severity was available, the current study considered any AEs requiring medical services. Assuming grade 3 or 4 AEs, that is, AEs typically included in economic modelling, would require medical services, these events would be captured in the current study. However, lower grade AEs may have also been captured if they required medical services. Third, claims data contain limited information on disease severity (e.g., cancer stage); as such, despite matching treatment episodes on an extensive set of observable characteristics, differences in unobservable characteristics (i.e., disease severity, course of disease) may remain. Lastly, for the analyses stratified by cancer type, the reliability and validity of the incremental healthcare cost estimates for AEs with low prevalence (e.g., pancreatitis) and certain cancers (e.g., skin) was limited by the small number of matched treatment episodes with AEs. In some cases, this may have resulted in negative cost estimates as a few outliers may drive the results. Negative cost estimates may also be explained by a high correlation between the studied AE and the presence of other AEs, where the cost of each AE could not be distinguished. Therefore, negative cost estimates should be interpreted with caution; negative estimates should not be interpreted as a cost saving, but rather as an indication of the potential presence of outliers or correlation in AEs associated with a low prevalence or estimates based on a limited sample size.

## Conclusions

This real-world study showed that, in the US, the AEs experienced by patients during cancer treatment were frequent and associated with a substantial economic burden. These results highlight the need for incorporating the assessment of potential AEs into the therapeutic decision-making process. Contrary to previous studies, which focused on specific AEs or cancer types, this study used a consistent methodology across multiple AEs and cancer types. Importantly, while some previous studies captured only the costs associated with the initial management of AEs, this study includes costs incurred both during and after the initial management phase, providing a more comprehensive estimate of the AE-related economic burden.
